# Metacognitive Treatment in Acquired Brain Injury and Its Applicability to Aphasia: A Systematic Review

**DOI:** 10.3389/fresc.2022.813416

**Published:** 2022-02-04

**Authors:** Amanda Wadams, Louisa Suting, André Lindsey, Jennifer Mozeiko

**Affiliations:** ^1^Department of Speech, Language and Hearing Science, University of Connecticut, Mansfield, MA, United States; ^2^School of Education, Speech Pathology, Nevada State College, Henderson, NV, United States

**Keywords:** metacognition, acquired brain injury, aphasia, rehabilitation, systematic reveiw

## Abstract

**Purpose:**

The purpose of this systematic review is to identify the utility of metacognitive therapeutic intervention for persons with acquired brain injury (ABI), with a focus on persons with aphasia.

**Methods:**

A search of six databases resulted in two hundred and sixty-six unique manuscripts relating to the explicit use of metacognitive treatment for people with ABI. Two independent reviewers rated abstracts for inclusion or exclusion of the study given predetermined criteria. Twenty-nine articles, five of which included people with aphasia, were selected for inclusion in this systematic review. SCED+ and PEDro+ rating scales were used to rate the methodological quality of each study.

**Results:**

Methodological quality of the 29 studies that met inclusion criteria ranged from weak to high quality studies. Three -hundred and sixty-nine individuals with ABI took part in the 29 studies. Varying treatment methods were employed. Outcome measures were inconsistent. Metacognitive treatment has been applied to people with aphasia with positive results, but efficacy of the treatment cannot yet be determined.

**Conclusions:**

Metacognitive therapeutic intervention tends to be effective for persons with acquired brain injury (ABI) despite variability between intervention designs and treatment outcomes across studies. Due to so few studies with participants with aphasia, we were unable to draw conclusions regarding the efficacy of metacognitive treatment for people with aphasia. Further research on the efficacy of metacognitive treatment for this population is warranted.

## Introduction

Metacognition is self-regulated insight into one's own thinking. It enables analysis and adjustments to be made in response to active behavioral performance as well as to changes in internal states. Metacognitive skills comprise two dynamic facets: metacognitive knowledge and online awareness (1). The former refers to judgement and understanding of one's ability to complete a task, whereas the latter is active engagement when carrying out a task (1). Metacognitive deficits are common following acquired brain injury (ABI), altering behavioral performance and negatively influencing safe engagement in independent activties of daily-living (IADLs) (2, 3).

Anosognosia, an unawareness of deficit (4), is often researched separately from metacognition. Though similar, the theoretical construct of the two were developed independently from one another (5). Sunderaraman and Cosentino (4) posit that metacognition explains more of the cognitive construct of unawareness, where anosognosia describes the clinical construct. If we were to fit anosognosia into the model of metacognition, it would be synonymous with the metacognitive knowledge facet (6). In this paper, we focus our research on the more all-ecompassing metacognition rather than anosognosia. The purpose of this systematic review is to identify the utility and effectiveness of metacognitive therapeutic interventions with individuals with ABIs including persons with aphasia.

In regards to typical aging, there is evidence that the dorsal and ventral white matter tracts atrophy, affecting both language and cognitive processing (7). There is evidence that the effect is more prominent in fluid cognition (ie. executive function or working memory) than crystallized cognitive abilities [recalling stored knowledge or past experiences; (8–10)]. That is, one will see less of an effect on tasks that rely on existent knowledge as compared to new tasks that rely on learning (10). Individuals with ABI rely on new learning; this finding implies increased difficulty in rehabilitation of older individuals with ABI. Metacognitive skills in older adults, which may be affected as a result of aging, can help account for the cognitive declines observed with age (11). Metacognitive training used to increase new learning and overall cognitive functioning in typical older adults has proven successful, which provides evidence that metacognitive rehabilitation for individuals with ABI may be successful as well.

A brain injury often times leads to cognitive deficits beyond aging in those affected. Following the brain injury, individuals remember themselves prior to the brain injury and may not grasp changed status in cognition. Metacognitive deficits following injury, in part, reflect a failure to update this knowledge in response to injury (i.e., recognition of current level of functioning) (1). Reduced ability to regulate these processes results in reduced success with completing tasks and can trigger implementation of maladaptive strategies. A failure to update knowledge results in overestimating performance abilty, which can lead to a sense of loss of control, depression, and isolation (1). In order to increase awareness of deficits, metacognitive knowledge, and online processing must be rehabilitated with individuals receiving care responsonible for identifying errors as they occur (1).

Metacognitive deficits are a common sequelae of ABI and reflect altered executive processes. Common areas of impairment include: initiation, flexibility, problem solving, self-monitoring, and self-regulation (12–14). Executive dysfunction can lead to significant life challenges including an inability to identify goals, pursue goals, apply learned strategies to different situations, and function independently within daily environment (12, 15, 16). Furthermore, deficits within these areas are also observed in language processing such as an inability to plan what to say, decreased success in delivering the message, and reduced ability with respect to inhibiting unwanted responses (17–19).

### Language

Aphasia, a common secondary result of ABI, is a multimodal language disorder in which the manipulation, comprehension and formulation of linguistic symbols and elements present as the prominent deficit in individuals affected (20–22). Though the primary deficit in aphasia is language, researchers have also identified concomitant impairments in working memory, self-regulation, attention, and executive function (23–25). Attentional skills are integrated in different stages of word production tasks including phonological encoding and lexical retrieval, and attentional skills required for these tasks can be affected in persons with aphasia (26, 27).

Types of aphasia can be broadly categorized into fluent and nonfluent aphasia; though metacognitive skills may be disrupted differently in each, lack of awareness may be present. Levelt et al. (28) proposed the perceptual loop hypothesis, stating that language output was monitored by one's comprehension of language. Therefore, if one does not comprehend errors, they would not be able to recognize and correct errors. The theory accounts for decreased metacognitive skills in those with fluent aphasia. Contrast, metacognitive awareness is postulated to be a conscious experience relying on both attention and executive function (29). Since these cognitive skills rely on the integrity and connections within the frontal cortex (17, 24), awareness in those with nonfluent aphasia may be affected. Evidence shows cognitive control and monitoring are important for word selection tasks and may be interrupted due to infarcts linked with aphasia (30). Moreover, performance on measures of awareness do correlate with severity of language impairment (6). Enhancing metacognitive skills is therefore likely to aid general cognitive functioning, subsequently bolstering linguistic performance.

### Rehabilitation

Metacognitive Strategy Instruction (MSI) involves training individuals to increase self-awareness of their strengths and weaknesses, thereby increasing their independence in completing everyday tasks. When describing treatment protocols that promote self-awareness, (31) state that self awareness retraining, “promote(s) internalization of self-regulation strategies through self-instruction and self-monitoring as a practice option” (p. 1688). In theory, once one is aware of strengths and weakness, they will be able to allocate resources where necessary (be it language or underlying cognitive deficits), thus increasing overall functioning. Another avenue for metacognitive training is a focus of error awareness throughout strategy training. The focus of this type of metacognitive training is the ability to recognize errors throughout completion of a task (14, 32). Once one has a heightened sense of awareness, they will be able to self-correct errors during language output.

Metacognitive therapy is used with individuals with traumatic brain injury (TBI) to increase self-awareness, self-reliance, and overall independence (33, 34). In this population metacognitive strategies commonly consist of breaking down goals into manageable steps, learning to change behavior to reach desired goal, and carrying out the change in behavior (35). Kennedy et al. (35) completed a meta-analysis evaluating therapeutic interventions for problem-solving, planning, organization, and multi-tasking in persons with TBI. The therapy dosage varied with each study, but all resulted in positive outcomes within a week post-treatment in various cognitive tasks with a trend of positive maintenance and generalization outcomes. The authors concluded that utilizing MSI for persons with TBI increased overall problem-solving skills.

Recent literature has emerged regarding the use of metacognitive therapy with people with aphasia (18, 25, 36–38). When provided during rehabilitation of cognitive-linguistic skills, metacognitive therapies are intended to enhance self-awareness and promote greater cognitive understanding and control during IADLs. Targeted skill sets include: the ability to set goals, evaluate performance throughout a task in relation to goals, decide how to change behavior in order to meet goals, and how to apply behaviors to new strategies in order to reach the desired outcome (39).

### Aims

The purpose of this systematic review is to identify the therapeutic effect of using metacognitive intervention for individuals with ABI, including persons with aphasia. There are four research objectives: (1) Describe and appraise the studies and the methodological quality of the studies reviewed (2) investigate whether metacognitive interventions result in positive outcomes (cognitive, language, social) for persons with ABI (3) determine whether there is a specific type of metacognitive intervention that is more widely utilized for individuals with ABI within the research literature (4) explore whether metacognitive intervention is or has the potential to be effective for persons with aphasia given extent and quality of the current literature.

## Methods

### Selection of Articles

Articles were selected from six electronic databases, including PubMed, Scopus, Linguistics and Language Behavior Abstracts (LLBA), American Speech Language and Hearing Association Journals (ASHA Journals), PsychInfo and ProQuest. An initial search of the databases was completed June 2018, with an updated search completed October 2019. Reference lists of identified studies were reviewed to identify studies that did not show up in the database search. Preferred Reporting Items for Systematic Reviews and Meta Analysis Guidelines [PRISMA, (40)] was employed. Keywords were: “metacognitive”; “online awareness” AND “treatment”; “intervention”; “rehabilitation” AND “aphasia”; “acquired brain injury”; “stroke”. Deduplication and screening were performed manually.

### Eligibility Criteria

In order to identify research articles appropriate for this systematic review, parameters for inclusion and exclusion criteria were set and included: full-text, peer-reviewed journal article in English, describing a completed metacognitive behavioral treatment published; original data from the study had to be reported; participants of interest were adults, over the age of 18, with a history of ABI, including penetrating head injury traumatic brain injury (PHI TBI), closed-head injury traumatic brain injury (CHI TBI), hypoxia, CVA, tumor, anoxia, arterial venous malformation, encephalitis or aneurysm.

We included only manuscripts that used treatments specifically designed to increase aspects of metacognition in participants, such as error detection, self-awareness, online awareness and the ability to identify and carry out appropriate compensatory strategies during a given task. Studies that sought to identify metacogntive deficits but did not explicity treat metacognition either directly or indirectly, were excluded. In this case, the researchers were looking for treatments explicitly targeting metacognition. For example, a study that treated attention as a primary outcome but that included metacognition as a secondary outcome would be included [i.e., (18, 37)] whereas one that only identified individuals with a metagcognitive deficit during the treatment process but did not track change, would have been excluded. Other articles excluded from the systematic review include: non-behavioral treatment studies such as those that use medication; studies including participants with a diagnosis of a degenerative disease (i.e., dementia). Gray literature and non-experimental publications (i.e., reviews) were excluded.

Three reviewers, the first, second, and fourth authors, completed initial parsing of the initial 266 journal articles based on the appraisal of the title and abstract of each paper included in the search results. If reviewers were not able to determine the eligibility of the paper based solely on the title and abstract, the full text was reviewed individually by two separate reviewers. Any disagreements between reviewers were brought to consensus through discussion.

### Methodological Quality Review

In order to identify the methodological quality of the studies included in the systematic review, the Physiotherapy Evidence Database Rating Scale-Plus (PEDro+) and the Single Case Experimental Design Scale-Plus (SCED+) were utilized (41). The PEDro was chosen due to its reliability in evaluating the quality of randomized control trials, including evaluation of the study's internal validity and adequacy in communicating interpretable statistical results (42). The SCED was chosen as a reliable quality measure of single subject research designs (43). The PEDro+ and SCED+ designs, as amended by Cherney et al. (41), were utilized in order to account for each study's treatment fidelity and treatment replicability in addition to the original quality measures.

Four reviewers, the authors of this manuscript, extracted data and completed the PEDro+ and SCED+ quality ratings. For reliability, each study was independently assessed by two reviewers. Upon reviewer disagreement of the rating score, further review of the article followed by discussion resulted in rater consensus.

### Data Extraction

The following data were systematically extracted from each article: year of publication, number of participants, age and gender of participants, type and severity of brain injury, time post onset of brain injury of each participant, concomitant diagnoses of the participants, whether the study utilized a control measure, design of the study, treatment type utilized in the study, duration of the intervention, and whether or not home practice was required as part of the study. The following outcome measures were obtained: cognitive, language, and rating scale outcome measures as well as any reports of the maintenance and/or generalization of the outcome skills. The clinical implications, study conclusions, study limitations and future research directions were also collected from each research article.

## Results and Discussion

*Aim 1: Describe and appraise the studies and the methodological quality of the studies reviewed*

### Literature Retrieved

**Figure 1:**
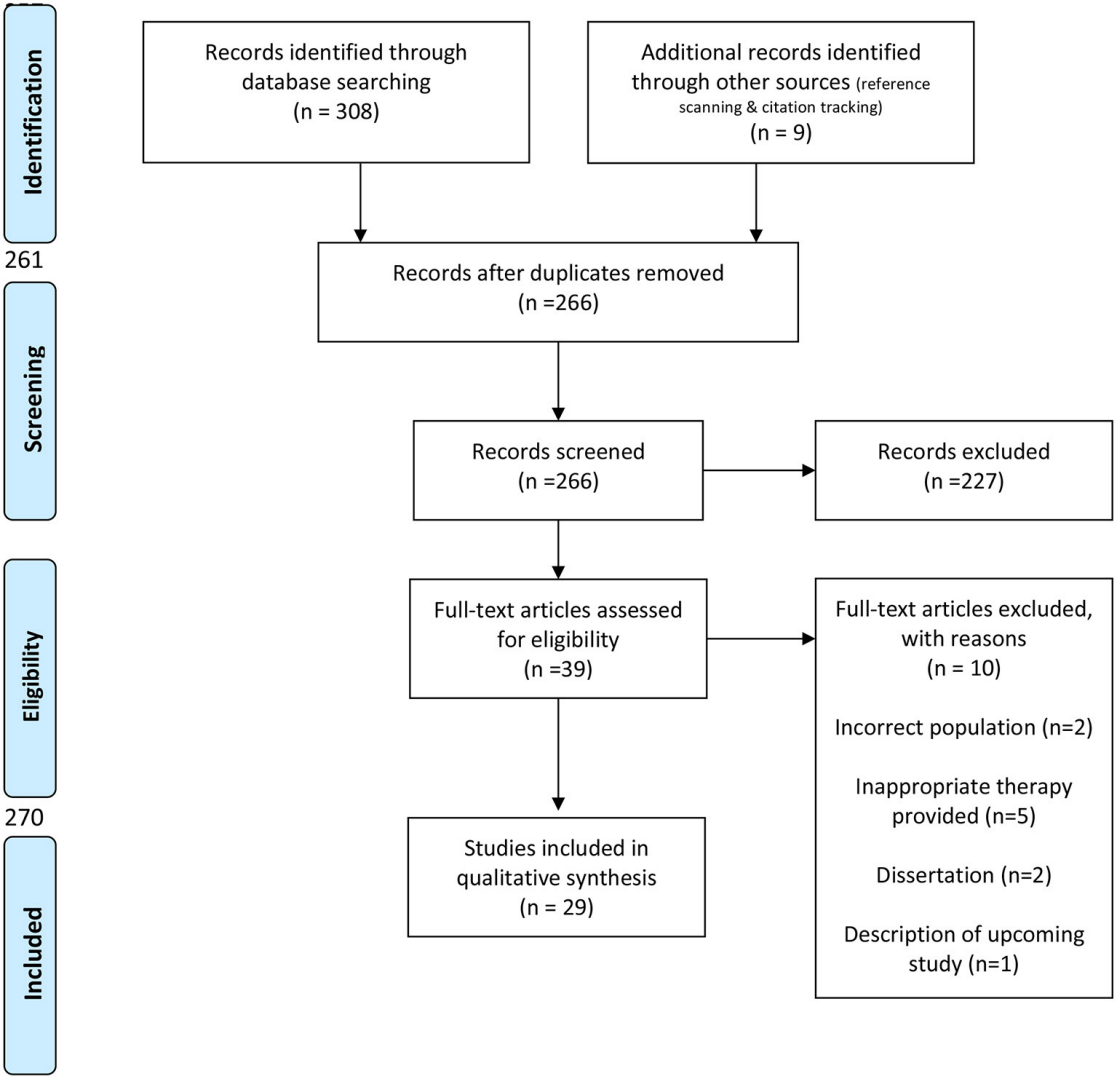
Prisma flow diagram.

The database search produced 257 articles following manual removal of duplicates. Through reference scanning and citation tracking, nine additional articles were determined to fit the study criteria, resulting in a total of 266 studies. Two-hundred and twenty-seven articles were removed following title and abstract screening. The remaining 39 articles underwent full article review, and ten additional articles were excluded due to inappropriate sample population [e.g., (44, 45)], treatment methods that did not involve metacognitive aspects [e.g., (46–49)], lack of peer review [e.g., (50–52)], or reporting of upcoming studies [e.g., (53)]. Of the 28 remaining articles, five were designed for people with aphasia. See Figure 1 for the PRISMA flow diagram, illustrating study selection.

In all, 29 studies were found to fit the criteria for metacognitive intervention. Twenty-eight of the 29 studies reported positive outcome measures on at least one of the measures utilized.

### Methodological Quality Rating

Six studies were reviewed using the PEDro+ rating scale and 24 studies were reviewed with the SCED+ rating. Levine et al. (54) included both a randomized control trial as well as a single subject design, so each was rated by the appropriate scale. Point by point interrater reliability was calculated for raters using the PEDro+ and the SCED+ scales, the interrater reliability scores were 93.65% and 95.24%, respectively. In order to be considered of adequate quality, a study must receive credit for at least half of the items on the checklist (41).

The results of the PEDro+ rating scale for each study are shown in Table 1. The methodological quality of the studies ranged from weak to high quality studies with scores ranging between six and eleven (out of a maximum of 13) (41). Five out of six RCTs received high ratings in this sample. On the PEDro+ rating scale, studies often lacked concealed allocation, blinding of therapists, blinding of assessors, and description of treatment fidelity.

**Table 1:** Pedro+ rating scale.

**References**	**Adequate clinical history^a^**	**Eligibility criteria specified^b^**	**Random allocation^c^**	**Concealed allocation^d^**	**Groups similar at baseline^e^**	**Blinding of subject^f^**	**Blinding of therapists^g^**	**Blinding of assessors^h^**	**Measure of at least one key outcome from more than 85% of subjects^i^**	**Intention to treat^j^**	**Between group statistical comparison^k^**	**Point measures/measures of variability^l^**	**Treatment fidelity^m^**	**Treatment replicability^n^**	**Total score**
Goverover et al. (55)	1	1	1	0	1	1	0	0	1	1	1	1	0	1	9
Levine et al. (54) (study 1)	1	1	1	0	1	0	0	0	1	1	1	1	0	1	8
Schmidt et al. (32)	1	1	1	1	1	1	0	1	1	1	1	1	0	1	11
Schmidt et al. (14)	1	1	1	1	1	0	0	0	1	1	1	0	0	1	8
Tornas et al. (56)	1	1	1	1	1	1	0	1	1	1	1	1	0	1	11
Villalobos et al. (57)	1	1	1	0	1	0	0	0	1	0	1	1	0	0	6

*PEDro rating scale utilized for rating randomized controlled trials. Rating of 1 denotes studies met specified criteria*.

a *Inclusion criteria must be specified*.

b *Eligibility criteria denotes that inclusion criteria is specified*.

c *Random allocation refers to that allocation to groups (experimental/control) is random*.

d *Concealed allocation refers to the idea that the person who determines eligibility for inclusion is unaware of group allocation*.

e *A statement must be made regarding group similarity at baseline*.

f *Subject blinding requires participants to be unaware of the group they are in*.

g *Therapist blinding requires that the therapist is not aware if they are delivering the experimental treatment*.

h *Assessor blinding means the outcome assessment is conducted by an individual who does not know whether or not the participant received the experimental treatment*.

i *Outcome measures must be measured for more than eight-five percent of participants*.

j *Intention to treat requires an explicit statement that all participants received the treatment or control condition to which they were allocated*.

k *Statistical comparison requires between group statistical comparisons be reported*.

l *Point measures and measures of variability are required to be provided for at least on key component of treatment*.

m *Treatment fidelity requires a report of adherence to the treatment protocol*.

n *Treatment replicability requires that the treatment process is clearly described or made availabl*.

The results of the SCED+ rating scale for each study are shown in Table 2. Scores on the SCED+ ranged from three to 12 (out of a maximum of 12). Sixteen out of 24 single case series designs achieved a high rating on the SCED+. In the SCED+, methodological quality ratings tended to be negatively impacted due to lack of interrater reliability, independence of assessors, and description of treatment fidelity.

**Table 2:** SCED-plus rating scale.

**References**	**Adequate clinical history^a^**	**Definition of target behaviors^b^**	**Experimental control of research design^c^**	**Baseline data^d^**	**Sampling of behaviors^e^**	**Raw data reported^f^**	**Inter-rater reliability^g^**	**Independence of assessors^h^**	**Statistical analysis^i^**	**Treatment replicated across participants^j^**	**Generalization^k^**	**Treatment fidelity^l^**	**Treatment replicability^m^**	**Total score (out of 13)**
Copley et al. (39)	1	1	1	0	0	1	0	0	1	1	1	1	1	8
Dawson et al. (58)	1	1	1	0	0	1	0	0	0	1	1	0	1	6
Finch et al. (59)	1	1	1	1	0	1	0	1	1	1	1	1	1	10
Fong and Howie (34)	1	1	1	0	0	1	0	0	1	1	0	0	1	6
Fitzgerald et al. (33)	1	1	0	0	0	1	0	0	1	1	0	0	1	5
Gilmore et al. (36)	1	1	1	0	0	1	0	0	1	1	1	1	1	8
Goodwin et al. (12)	1	0	1	1	0	0	0	0	1	0	0	0	1	4
Laatsch and Stress (60)	1	1	1	0	0	1	0	1	1	1	0	0	1	7
Laatsch et al. (61)	1	0	1	0	0	0	0	0	1	0	0	0	0	2
Lee and Sohlberg (18)	1	1	1	1	1	1	0	0	1	1	1	0	1	9
Lee et al. (37)	1	1	1	1	1	1	1	1	1	1	1	0	1	11
Levine et al. (54) (study 2)	1	1	1	0	0	1	0	0	0	0	0	0	1	4
Mayer et al. (25)	1	1	1	0	0	0	0	0	0	0	1	0	1	4
Novokovic-Agopian et al. (13)	1	1	1	0	0	1	0	1	1	1	1	0	1	8
Ownsworth et al. (2)	1	1	1	1	1	1	1	0	0	0	1	0	1	8
Ownsworth et al. (3)	1	1	1	1	1	1	1	0	1	1	1	0	1	10
Ramanathan et al. (62)	1	1	1	1	1	1	0	0	1	0	1	1	1	9
Raskin et al. (63)	1	1	1	0	0	1	0	0	1	1	1	0	1	7
Rosell-Clari and Hernandez-Sacristan (38)	1	1	1	0	0	0	0	0	1	0	0	0	1	4
Skidmore et al. (15)	1	1	1	0	1	1	0	0	0	0	1	0	1	6
Toglia et al. (64)	1	1	1	0	0	1	0	0	0	0	1	0	1	5
Toglia et al. (64)	1	1	1	0	0	1	0	0	0	1	1	0	1	6
Waid-Ebbs et al. (16)	1	1	1	1	0	0	0	0	1	1	1	0	1	7

*SCED-Plus rating scale used for rating single subject design studies. Rating of 1 denotes studies met specified criteria*.

a *Clinical history requires sufficient description of the participant including age, etiology, TPO and severity*.

b *Target behaviors of each participant are required to be operationally defined*.

c *The research design needed to be deemed to meet sufficient experimental control*.

d *Baseline data requires that behviors were sufficiently measured prior to initiation of treatment (3 stable point measures)*.

e *Sampling of behaviors required at least every other session*.

f *Raw data to be reported, be it in graphs or tables*.

g *Inter-rater reliability report is required*.

h *Independence of assessors requires the individual assessing outcome measures not be the individual implementing treatment*.

i *Statistical analysis required to be reported*.

j *Treatment required to be replicated across participants*.

k *Generalization beyond training condition should be reported*.

l *Treatment fidelity requires a report of adherence to the treatment protocol*.

m *Treatment replicability refers to the idea that the treatmet process is clearly described or made available*.

*Aim 2: investigate whether metacognitive interventions result in positive outcomes (cognitive, language, social) for persons with ABI*

### Study Characteristics

#### Population

Table 3 describes the characteristics of participants included in each study. Three-hundred and seventy individuals with ABI took part in the 29 aforementioned studies. ABI severity ranged from mild to severe; ages ranged from 18 to 83; and all had at least an 8th grade education. Etiology included: TBI (CHI and PHI), hypoxia, CVA, tumor, anoxia, arterial venous malformation, encephalitis, and aneurysm. Time post onset (TPO) of the injury ranged from 1 week to 34 years. Concomitant impairments included dysarthria, right hemianopsia, anxiety, depression, hearing loss, amnesia, hemiparesis or paralysis, and apraxia of speech.

**Table 3:** Population characteristics.

**References**	**# of participants**	**Gender**	**Age**	**Education (yrs)**	**TPO (mos)**	**Etiology**	**Severity**
Copley et al. (39)	8	5 male, 3 female	25–70 (M = 40.75)	10–16 (M = 12.75)	4–21 (M = 12)	TBI & hypoxic ABI	Moderate to severe
Dawson et al. (58)	3	2 male, 1 female	32–43 (M = 38.33)	14–17 (M = 16)	60–240 (M = 168)	TBI	Mild to severe
Finch et al. (59)	8	4 male	23–49 (M = 36.25)	High school +	4–56 (M = 24)	TBI	Mild to severe
Fitzgerald et al. (33)	6	5 male, 1 female	20–34 (M = 27.2)	NR	3–223 (M = 87.02)	TBI	Severe
Fong and Howie (34)	16	12 male, 4 female	M = 30.6	M = 10.5	M = 11.8	TBI, intracerebral hemorrhage, tumor, arterial-venous malformation, encephalitis	Moderate
Gilmore et al. (36)	4	4 male	21–34 (M = 27.25)	12–16 (M = 13.75)	49–97 (M = 78)	TBI & CVA	Mild to severe
Goodwin et al. (12)	66	41 male, 25 female	18–61 (M = 35.02)	NR	>1	TBI, CVA, aneurysm, anoxia, encephalitis, hypoxaemia	NR
Goverover et al. (55)	10	8 male, 2 female	M = 39.5	M = 13.2	M = 12.9	TBI	NR
Kintz et al. (65)	3	2 male, 1 female	M = 46.67	M = 13	M = 78	TBI	Mild to moderate
Laatsch and Stress (60)	37	14 male, 23 female	14–65 (M = 33.6)	8–20 (M = 13.6)	1–228 (M = 23.9)	TBI, CVA, tumor, anoxia, MS, seizure disorder	Mild to severe
Laatsch et al. (61)	1	Female	38	15	192	TBI	Mild to moderate
Lee and Sohlberg (18)	4	2 male, 2 female	57–83 (M = 71.25)	14–23 (M = 17.25)	18–79 (M = 43.25)	Left CVA	Mild to moderate
Lee et al. (37)	6	5 male, 1 female	56–66 (M = 61.5)	13–19 (M = 15.5)	9–80 (M = 44.17)	Left CVA	Mild
Levine et al. (54) (Study 1)	15	5 male, 10 female	M = 29	M = 12.6	M = 44.4	TBI	NR
Levine et al. (54) (Study 2)	1	Female	35	16	5	Meningo-encephalitis	NR
Mayer et al. (25)	1	Male	63	NR	4 mos	Left CVA	Mild to moderate
Novakovic-Agopian et al. (13)	16	7 male, 9 female	24–63 (M = 50.375)	16–19 (M = 16.625)	Chronic	TBI, stroke, leukoencephalopathy	Mild to moderate
Ownsworth et al. (2)	1	Male	≅34	10	≅24	PHI TBI	Severe
Ownsworth et al. (3)	3	2 male, 1 female	26–43 (M = 35.33)	NR	24–84 (M = 60)	PHI TBI, CHI TBI	Severe
Ramanathan et al. (62)	1	Male	54	>10th grade	90	CHI TBI	Moderate- severe
Raskin et al. (63)	20	12 male, 8 female	M = 42.11	M = 13.64	M = 217.19	ABI	Moderate to severe
Rosell-Clari and Hernandez Sacristan (38)	1	Female	Early 70s	12	≅2.5 yrs	Left CVA	NR
Schmidt et al. (32)	54	NR	M = 40	NR	M = 48	TBI	NR
Schmidt et al. (14)	10	7 males, 3 females	M = 44.7	M = 14.4	M = 31.2	TBI	Mild to severe
Skidmore et al. (15)	1	Male	31	12	7 days	Right CVA	Moderate to severe
Toglia et al. (64)	4	2 male, 2 female	27–50 (M = 38.25)	≥12	37–67 (M = 48.75)	TBI	NR
Toglia et al. (64)	1	1 female	29	12	M = 66	TBI	Moderate
Tornas et al. (56)	33	19 male, 14 female	M = 42.12	M = 13.23	M = 106.94	TBI, CVA, tumor	NR
Villalobos et al. (57)	30	20 male, 10 female	M = 40.37	M = 11.7	≅5	TBI, CVA, brain tumor, encephalitis, surgery, HIV	NR
Waid-Ebbs et al. (16)	6	4 male, 2 female	25–40 (M = 31.33)	NR	NR	TBI	NR

*CVA, Cerebral Vascular Accident; TBI, Traumatic Brain Injury; PHI TBI, Penetrating-Head Injury Traumatic Brain Injury; CHI TBI, Close-Head Injury Traumatic Brain Injury; ABI,Acquired Brain Injury; HIV, Human Immunodeficiency Virus; MS, Multiple Sclerosis*.

Though reports of patient characteristics were generally considered detailed, defining four or more characteristics regarding the participant, there were trends of missing characteristics important to patient history noted. The missing components tended to include site of lesion data, handedness, prior treatment history and vision and hearing status.

#### Research Design

A wide range of study designs are included in this systematic review. The most frequently used research designs among studies were repeated measures (12, 61, 64) and single subject study designs [see Table 4; (2, 3, 15, 18, 25, 37, 38, 54, 62)].

**Table 4:** Research & treatment designs.

**References**	**Experimental design**	**treatment program**	**Session duration (hours)**	**Session frequency (times per week)**	**Treatment duration (hours)**
Copley et al. (39)	ABA	MSI	1.5 (group) 2 (individual)	3	22
Dawson et al. (58)	Case Series	CO-OP	1	2	20
Finch et al. (59)	Cohort Study	MSI	1	2	16
Fitzgerald et al. (33)	RCT	CPT	0.67	2	5.3
Fong and Howie (34)	Controlled trial matched pairs	Metacomponential Skills Training	1.25	2	37.5
Gilmore et al. (36)	Quasi-experimental	ICCR	6	5	360
Goodwin et al. (12)	Repeated measures	OZC program	6–8	4	288–384
Goverover et al. (55)	Single blind RCT	Self-Awareness Retraining	0.75	2–3	4.5–6.75
Kintz et al. (65)	A–B	DPT	1	4	16
Laatsch and Stress (60)	Retrospective study	Developmental metacognitive approach	1	1–2	11–22
Laatsch et al. (61)	Repeated measures	Developmental metacognitive approach	1	3	96
Lee and Sohlberg (18)	Single subject	APT-3	0.5–0.75	4	16–24
Lee et al. (37)	Non-current multiple baseline SCED	APT-3	0.5–0.67	6	18–24.12
Levine et al. (54) (Study 2)	Single case study	GMT	NR	7 ×	NR
Levine et al. (54) (Study 1)	RCT	GMT	1	2 ×	2
Mayer et al. (25)	Single subject case study	Brain budget protocol	1	4–5 for 2 weeks; 2 × for 9 wks	26–28
Novakovic-Agopian et al. (13)	Pseudo-random cross over	Goal oriented attentional self-regulation training	2 (group) 1 (individual), 20 (home practice)	NR	43
Ownsworth et al. (2)	Single case experimental	MST psychological and socioenvironmental factors	NR	1	NR
Ownsworth et al. (3)	Single subject ABA	MST	1.5–2	8	12–16
Ramanathan et al. (62)	A–B	CRT, APT-III, PM Training	2.5	4	30
Raskin et al. (63)	AB–BA	Combinatorial	1	1–2	24–48
Rosell-Clari and Hernandez Sacristan (38)	Single subject experimental treatment study	Pragmatic functional paradigm	0.5	3	48
Schmidt et al. (32)	RCT with 3 intervention groups	Feedback groups	NR	2–3	NR
Schmidt et al. (14)	Prospective and longitudinal RCT	Feedback groups	NR	2	NR
Skidmore et al. (15)	Single case study	CO-OP	0.75	5	7.5
Toglia et al. (64)	Single subject with repeated measures	Multi-context approach	1.25	2	12.5
Toglia et al. (64)	Single subject with repeated measures	Multi-context approach	1.25	2	25
Tornas et al. (56)	RCT	GMT	2	10	16
Villalobos et al. (57)	RCT	AD treatment	NR	8 ×	NR
Waid-Ebbs et al. (16)	A–B	GMT	NR	2	NR

*RCT, Randomized Control Trial; MSI, Metacognitive Strategy Instruction; CPT, Continuous Performance Training; CO-OP, Cognitive Orientation to Daily Occupational Performance; ICCR, Intensive Cognitive-Communication Rehabilitation; OZC, Oliver Zangwill Center for Neuropsychological Rehabilitation; DPT, Discourse Processing Treatment; APT, Attention Process Training; GMT, Goal Management Training; MST, Metacognitive Skills Training; CRT, Cognitive Rehabilitation Therapy; PM, Prospective Memory; AD, Awareness of Deficit*.

#### Treatment Paradigm

Table 4 identifies treatment dosages, treatment types and treatment designs utilized across studies. Treatment dosages between and within treatment types were variable. Intensive treatment programs ranged from 4 to 8 h a day, spanned treatment durations of 4 days up to 12 weeks. The dosage of intensive treatment protocols ranged greatly from as few as 16 h over 4 days/2 weeks (56) up to 369 h over 5 days/12 weeks (36) of active treatment.

A wide range of treatment protocols with a focus on increasing and utilizing metacognitive skills were also used across studies. In each, the metacognitive treatment was either implemented as a standalone treatment or as a concurrent treatment with cognitive or language-based treatments such as: Attention Processing Training-III [APT-III; Lee and Sohlberg (37)], cognitive rehabilitation therapy [CRT; (12, 14, 32, 62)] or pragmatic language functioning (38). The treatments also varied between individual treatment, group treatment or a hybrid of both. In each treatment included in the systematic review, the goal was to increase an individual's self-awareness while simultaneously training the individual in the use of compensatory strategies to increase independence. A total of 21 different treatment paradigms were utilized across studies (see Table 4), though there were many patterns seen across treatment paradigms. The most commonly utilized metacognitive treatment paradigms, included Metacognitive Strategy Instruction (MSI), Goal Management Training (GMT), Cognitive Orientation to Occupational Performance Intervention (CO-OP), and Verbal and Video Feedback. See Supplementary Material A for more details on metacognitive treatment type.

### Outcomes

Outcome scores were recorded in three areas: rating scale outcomes (Table 5), cognitive assessment outcome measures (Table 6) and language outcome measures (Table 7). Positive changes in treatment were defined by authors of each paper and reported accordingly in this review.

**Table 5:** Rating scale outcomes.

**Study**	**Treatment program**	**Rating scale measures**	**Positive outcomes observed**
**Self-perception of cognition rating scales**			
Dawson et al. (58)	CO-OP	COPM, DEX	Yes^*^
Finch et al. (59)	MSI	GAS	GAS: yes
Fitzgerald et al. (33)	CPT	FRsBe, PCRS, CFQ	FrsBe: yes PCRS: no CFQ: yes
Fong and Howie (34)	Metacomponential skills training	MI	MI: yes
Gilmore et al. (36)	ICCR	GAS	Yes^*^
Goodwin et al. (12)	OZC program	DEX & DEX-1	DEX/DEX-1: yes
Goverover et al. (55)	Self-awareness retraining	SRSI, AQ	SRSI: yes AQ: no
Novakovic-Agopian et al. (13)	Goal oriented attentional self-regulation training	Goal processing questionnaire	Yes^*^
Ownsworth et al. (2)	MST with psychological and socioenvironmental factors	SADI, AQ	No
Ownsworth et al. (3)	MST	PCRS	Yes^*^
Raskin et al. (63)	Combinatorial	PMQ, EMQ	PMQ: no EMQ: yes
Schmidt et al. (32)	Feedback groups	AQ, SPIRQ	AQ: yes SPIRQ: no
Schmidt et al. (14)	Feedback groups	AQ	AQ: yes
Skidmore et al. (15)	CO-OP	COPM	COPM: yes^*^
Toglia et al. (64)	Multi-context approach	AQ, BRIEF-A, SRSI	SRSI: yes^*^ AQ: no BRIEF-A: no
Toglia et al. (64)	Multi-context approach	SRSI, BRIEF-A, AQ	AQ: no SRSI: no BRIEF-A: yes
Tornas et al. (56)	GMT	BRIEF-A, CFQ, DEX	BRIEF-A: yes CFQ: yes DEX: yes
Villalobos et al. (57)	AD treatment	Awareness of injury, awareness of deficit and awareness of disability scales	Awareness of Injury: yes Awareness of Deficit: yes Awareness of disability: yes
Waid-Ebbs et al. (16)	GMT	BRIEF-A	BRIEF-A: no
**Communication rating scales**			
Finch et al. (59)	MSI	PPIC, LCQ	PPIC: yes LCQ: no
**Quality of life rating scales**			
Gilmore et al. (36)	ICCR	TBI-QOL, Neuro-QOL, CASP	Yes^*^
Goverover et al. (55)	Self-awareness retraining	CIQ	CIQ: yes
Raskin et al. (63)	Combinatorial	WHO-QOL	WHO-QOL: yes
Schmidt et al. (32)	Feedback groups	DASS	DASS: no
Schmidt et al. (14)	Feedback groups	DASS	DASS: no

*CO-OP, Cognitive Orientation to Daily Occupational Performance; MSI, Metacognitive Strategy Instruction; CPT, Continuous Performance Task; ICCR, Intensive Cognitive Communication Rehabilitation; OZC, Oliver Zangwill Center for Neropsychological Rehabilitation; MST, Metacognitive Skills Training; GMT, Goal Management Training; AD, Awareness of Deficit; COMP, Canadian Occupational Performance Measure; DEX, Dysexecutive Questionnaire; PPIC, Profile of Pragmatic Impairment in Communication; GAS, Goal Attainment Scaling; LCQ, LaTrobe Communication Questionnaire; FRsBE, Frontal Systems Behavior Scale; PCRS, Patient Competency Rating Scale; CFQ, Cognitive Failures Questionnaire; MI-Metacomponential Interview; CASP, Child and Adolescent Scale of Participation; TBI-QOL, Traumatic Brain Injury Quality of Life; Neuro-QOL, Neurologic Quality of Life; SRSI, Self-regulation Skills Interview; AQ, Awareness Questionnaire; CIQ, Community Integration Questionnaire; SADI, Self-Awareness of Deficits Interview; PMQ, Prospective Memory Questionnaire; EMQ, Everyday Memory Questionnaire; WHO-QOL, World Health Organization Quality of Life; DASS, Depression Anxiety Stress Scale; SPIRQ, Self-perceptions in Rehabilitation Questionnaire; BRIEF-A, Behavior Rating Inventory of Executive Function-Adult Version*.

*Effect Sizes- provided by researchers or calculated by author when there were adequate baseline and follow-up data points*.

* *Researchers denote statistically significant change in research article*.

**Table 6:** Cognitive measure outcomes.

**References**	**Cognitive measures**	**Positive changes observed**
Fitzgerald et al. (33)	DART EAT	DART: yes EAT: no
Fong and Howie (34)	RPM, BADS key search, MEPSM, SPSVM	RPM: no BADS: no MEPSM: no SPSVM: no
Gilmore et al. (36)	RBANS, classroom behavior, SCCAN	RBANS: mixed Behavior: yes SCANN: yes^*^
Goverover et al. (55)	AAD	AAD between groups: no
Laatsch and Stress (60)	WAIS-R IQ (IQ), Stoop Color Inference (ProSp), WCST Problem Solving (probsolv) WMS-R: Verbal Immediate Memory Test (VerMemST), Verbal Delayed Memory Test (vermemlt),Visual Immediate Memory Test (vermemst) Visual Delayed Memory Test (vermemlt)	WAIS IQ: yes ProPp: yes probsolv: yes verbmenst: yes verbmemlt: yes vismemst: yes vismemlt: yes
Laatsch et al. (61)	Trails A, Trails B, digit vigilance test speed, digit vigilance test errors, letter verbal fluency, rey complex figural design immediate, rey complex figural design delayed	Yes^*^
Lee and Sohlberg (18)	CPT-II, TEA	Mixed
Lee et al. (37)	CPT-II, TEA Map Search, WMS-III Spatial Span, PALPA Span for Verb-Noun Sequences, TEA Visual Elevator, TEA Telephone Search Dual Task	CPT-II: no TEA Map Search: yes TEA Visual Elevator: no TEA Dual Task Decrement: no WMS Spatial Span: no PALPA Span: yes
Levine et al. (54) (Study 2)	Error frequency on paper and pencil tasks & meal preparation task	Yes
Levine et al. (54) (Study 1)	Errors and speed on given paper and pencil tasks (proofreading & grouping)	Yes
Novakovic-Agopian et al. (13)	Auditory Consonant Trigrams, WAIS III Letter Number Sequencing, Digit Vigilance Test, DKEFS: Stroop Inhibition-Switching, Design Fluency Switching, Verbal Fluency Switching; HVLT-R, BVMT-R, MET, Trails A & Trails B	Yes^*^
Ownsworth et al. (2)	Error frequency and error behavior	Yes^*^
Ownsworth et al. (3)	Error behaviors, checks, self-corrected errors and therapist-corrected errors	Yes^*^
Ramanathan et al. (62)	D-KEFS, APT-III, MIST	Yes^*^
Raskin et al. (63)	MIST, Trail Making Test, Brief Test of Attention, HVLT	MIST: yes^*^ Trail Making A: no Trail Making B: yes^*^ Brief Test of Attention: yes^*^, HVLT total recall: no
Schmidt et al. (32)	Number of errors	Yes
Schmidt et al. (14)	Error frequency	Yes^*^
Toglia et al. (64)	EFPT bill paying task & MET	Yes^*^
Toglia et al. (64)	EFPT bill paying task & MET	Yes
Tornas et al. (56)	CPT-II, DKEFS: CWI, VFT 3, Tower Test, TMT; Hotel Task, UPSA	Yes
Waid-Ebbs et al. (16)	TOL	Yes

*DART, Dual-Task Attention Response Task; EAT, Error Awareness Task; RPM, Raven's Progressive Matrices; BADS, Behavioral Assessment of the Dysexecutive Syndrome; MEPSM, Means-Ends Problem Solving Measure; SPSVM, Social Problem Solving Video Measure; RBANS, Repeatable Battery for Assessment of Neuropsychological Status; SCCAN, Scales of Cognitive and Communicative Ability for Neurorehabilitation; AAD, Assessment of Awareness of Disability; WAIS-R, Weschler Adult Intelligence Scale-Revised; IQ, Intelligence Quotient; WCST, Wisconsin Card Sorting Test; WMS-R, Weschler's Memory Scale-Revised; CPT, Connor's Continuous Performance Test; TEA, Test of Everyday Attention; PALPA, Psycholinguistic Assessments of Language Processing in Aphasia; DKEFS, Delis-Kaplan Executive Function System; MET, Multiple Errands Task; HVLT-R, Hopkins Verbal Learning Test-Revised; BVMT-R, Brief Visual Memory Test-Revised; MIST, Memory for Intentions Screening Test; CWI, Color-Word Interference Test; VFT, Verbal Fluency Test; UPSA, UCSD Performance Based Skills Assessment; TMT, Trail Making Test; TOL, Tower of London*.

Effect Sizes- provided by researchers or calculated by author when there were adequate baseline and follow-up data points.

* *Researchers denoted statistically significant change in research article*.

**Table 7:** Language outcome measures.

**References**	**Language measures**	**Positive changes observed**
Copley et al. (39)	MCLA	Yes
Gilmore et al. (36)	WAB-R, DCT	WAB: yes DCT: no
Kintz et al. (65)	Thematic units	Mixed
Laatsch et al. (61)	Woodcock-Johnson reading comprehension, iowa reading test	W-J Reading Comprehension: no Iowa Reading Test: no
Lee and Sohlberg (18)	AIMSWeb maze reading	Mixed (2/4 participants)^*^
Lee et al. (37)	Maze reading	Yes
Mayer et al. (25)	Oral reading, verbal expression, written expression	Oral reading: yes^*^ verbal expression: yes^*^ written expression: no
Ramanathan et al. (62)	ASHA FACS	Pre-test WNL
Rosell-Clari and Hernandez-Sacristan (38)	BDAE MetAphAs	BDAE: no MetAphAs: mixed

*MCLA, Measure of Cognitive Linguistic Abilities; WAB-R, Western Aphasia Battery-Revised; DCT, Discourse Comprehension Test; ASHA FACS, American Speech, Language and Hearing Association's Functional Assessment of Communication; BDAE, Boston Diagnostic Aphasia Examination; MetAphAs, MetaLanguage in Aphasia Assessment*.

*Effect Sizes- provided by researchers or calculated by author when there were adequate baseline and follow-up data points*.

* *Researchers denoted statistically significant change in research article*.

#### Rating Scale Outcomes

In order to capture metacognitive changes in participants, self-report questionnaires were utilized; questionnaires were completed by either the participant themselves or a caregiver. Table 5 identifies rating scales used in each study as well as the results observed in each treatment study. Results varied within and between rating scales across studies, revealing either no significant change in metacognitive skills pre to post treatment to significant positive changes in metacognitive functioning. See Supplementary Material B for further details.

#### Cognitive Testing Outcomes

To identify cognitive outcomes in participants, an array of assessments were utilized and included tests of attention, executive function, problem solving, visual and verbal memory, task completion, error awareness, and error frequency as well as visual scanning. A complete list of cognitive tests utilized in the studies can be found in Table 6. The most widely used tests were the Trails B, Connor's Continuous Performance Test-II (CPT-II), and error frequency.

Table 6 identifies immediate positive outcomes (within a week of cessation of treatment) of each of the studies. Of the 29 studies included in this review, 12 assessed maintainence of skills at least 1 month post treatment. Of the 12 studies, 11 studies reported maintainence of skills among participants (2, 3, 14, 54, 56, 58, 59, 62–65) and one study did not report maintance of skills among participants (16). See Supplementary Material C for further detail.

#### Language Testing Outcomes

Language outcome measures varied throughout studies (See Table 7 for list of measures utilized). The only measure that was used in more than one study was the AIM's maze reading measure utilized in a series of studies conducted by Lee and Sohlberg (18) and Lee et al. (37); the rest of the outcome measures were unique to each study. Measures also varied between standardized and non-standardized outcomes. The assessments ranged in what they were testing including expressive language, discourse measurements and reading comprehension. Metacognitive rehabilitation was shown to be effective for language in some studies (25, 39), but demonstrated mixed effects in other studies (18, 25, 36, 65) and were not effective in two studies (38, 61).

### Do Metacognitive Interventions Demonstrate Positive Outcomes for Persons With ABI?

The studies included in the final analysis utilized various research designs, therapeutic strategies, and assessment measures, making comparison difficult, but collectively they have provided insight regarding the use of metacognitive intervention with the ABI population. All 29 metacognitive interventions resulted in positive or mixed outcomes on rating scale, cognitive or language assessments (21 mixed outcomes and eight positive outcomes across scales). Of the studies measuring rating scale outcomes, eight of 19 studies had positive results, nine of 19 had mixed results between assessments, and two of 19 resulted in no change. Seventeen out of 19 studies utilizing rating scales observed at least one positive trend at completion. Nineteen out of 20 studies utilizing cognitive outcome measures reported at least one positive trend in cognitive measures following treatment that included metacognition. Fourteen of the 21 studies reported positive outcomes on all measures used, five out of the 21 studies had mixed outcomes between assessments [i.e, clinically significant change on the TEA Map Search, but not on CPT-II; (37)], and two out of the 21 studies reported no change on any outcome measure (34, 55). Similarly, seven out of nine metacognitive interventions resulted in at least one positive language outcome amongst measures.

Positive characteristics of metacognitive treatment include the feasibility and functionality of the treatment program, where participants can apply treatment to their everyday life (25, 58, 59). The programs are also flexible, allowing each treatment to be tailored to an individual's needs (25, 33, 38).

Authors also reported caveats of treatment protocols, including that the treatment can be taxing on cognitive skills that may be interrupted due to brain injury. The reliance on cognitive skills that may have been impacted due to injury may inhibit treatment outcomes due to the inability of individuals to apply learned material, thus making metacognitive treatment difficult for some. Inadequate length of treatment was also deemed to have had a negative effect on treatment outcomes, with authors suggesting that more time in treatment may have a more positive impact on effectiveness and generalizability of the treatment (55, 60). Considering these factors, metacognitive treatment for persons with ABI does appear to be effective, where positive changes across participants were observed on at least one measure in 22 out of 29 of the studies.

*Aim 3: determine whether there is a specific type of metacognitive intervention that is more widely utilized for individuals with ABI within the research literature*

MSI, GMT, CO-OP and APT-II are the only metacognitive interventions that were utilized in more than one study, however, no one of these appears to have been used more widely than others. On the other hand, there are some techniques that are more commonly used within each treatment paradigm. Goal setting and providing feedback on errors emerged as important components within treatment paradigms. MSI was utilized across two studies for individuals with mild to severe ABI (39, 59). Each study included eight participants where Finch et al. (59) utilized metacognitive intervention for social communication and Copley et al. (39) utilized MSI to address receptive language skills. Finch et al. (59) obtained mixed rating scale outcome measures (positive PPIC and GAS, no change seen onLCQ) and Copley et al. (39) identified positive language outcome measures.

GMT was utilized in three studies for individuals with ABI (severity unreported), with a total of 54 participants. Positive outcome measures were observed on both rating scale measures and cognitive measures (16, 54, 56).

CO-OP was utilized in two studies included in this systematic review; three participants were diagnosed with mild to severe TBI (58) and one participant was diagnosed with a moderate to severe right hemisphere stroke (15). Both research teams identified positive rating scale outcome measures upon completion of treatment.

APT-III was used to treat reading comprehension in ten individuals with mild to moderate aphasia in two studies and in both explicit feedback on performance was provided following each treatment session (18, 37). Findings on cognitive and language outcome measures were mixed between participants within these studies on both the TEA and Maze reading tasks. Two studies utilized feedback groups on persons with mild to severe TBI through completion of iADLs.

Although there is an inherent difference between self-awareness retraining (MSI, GMT, CO-OP) and error awareness training (Verbal and Video Feedback), there are many similarities between the two. In MSI, CO-OP, and GMT, breaking down the steps to completion and review of a task are still the main tenants; each of these metacognitive treatment paradigms include a review of success of task completion and errors. The self-awareness treatments also encourage participants to self-generate strategies to ensure successful task completion in the future, which implies recognizing errors. In Verbal and Video Feedback groups, participants were asked to rate their performance prior to initiating a task as well as following task completion. This strategy promotes self-awareness of abilities in addition to highlighting error awareness throughout the task. There is a cross-over between these two approaches, though each focuses different skills.

Several different interventions were utilized among the studies examined in this systematic review. GMT was the most commonly utilized and had consistently positive outcome scores compared to the other interventions. It was utilized in only three studies but included a relatively large number of participants (*n* = 54). Three studies are not sufficient to deem it the most effective type of treatment, but there is strong evidence supporting its use.

*Aim 4: Explore whether metacognitive intervention is or has the potential to be effective for persons with aphasia given extent and quality of the current literature*.

Though ABI includes individuals with aphasia following a stroke, those with different etiologies may respond differently to treatment. Only five of the 29 studies, with a total of 15 participants, focused on metacognitive rehabilitation for people with aphasia (18, 25, 36–38). Fourteen of the individuals with aphasia presented with mild to moderate aphasia, and one presented with severe aphasia. Three of the five studies focused on a combination of cognitive and language outcome measures (18, 36, 36) and two of the studies focused on language outcomes only (25, 66). Of the 15 participants, nine participants demonstrated positive results on language measures in response to metacognitive treatment, incuding increased attention, expressive language, and oral reading skills. With over half of the participants demonstrating a positive response to metacognitive treatment, one could make a case for implementing these aspects into language treatments. More research needs to be done to determine the optimal candidate with aphasia for this type of treatment.

Table 8 provides characteristics and outcomes from the studies focused on aphasia.

**Table 8:** Aphasia outcomes.

**References**	**# of participants (with aphasia)**	**Type of aphasia**	**Severity of aphasia**	**Type of treatment**	**Hours of treatment**	**Positive changes observed**
Gilmore et al. (36)	3	Broca's; unknown	Mild to severe with cognitive deficits	ICCR	360	Mixed
Lee and Sohlberg (18)	4	Anomic & conduction	Mild to moderate	APT-3	18–24	Mixed
Lee et al. (37)	6	N/A	Mild	APT-3	18–24.2	Mixed
Mayer et al. (25)	1	N/A	Mild to moderate	Brain budget protocol	26–28	Yes
Rosel-Clari and Hernandez-Sacristan (66)	1	Motor-mixed	NR	Pragmatic functional paradigm	48	Mixed

*ICCR, Intensive Cognitive-Communication Rehabilitation Program; APT, Attention Process Training; NR, Not Reported*.

### Is Metacognitive Intervention Effective for Persons With Aphasia?

Treatments of persons with aphasia focused on those diagnosed with nonfluent aphasia with a given severity ranging from mild to severe. The individual with severe aphasia did not show improvement on language measures, though more research should be completed to identify the optimal population for metacogntive treatment. No singular treatment protocol was used throughout studies; APT-III was the only treatment protocol used more than once in the studies included for analysis (18, 37).

Effectiveness of treatments varied across measures and participants within each study. Though some positive effects were observed by researchers, most treatments did not identify positive outcomes for every participant and/or on every measure used (including cognitive, language and rating scale outcome measures). Of the studies utilizing rating scale measures, three out of three participants demonstrated positive changes on quality of life and goal attainment measures (36). Of the studies utilizing cognitive measures in addition to language measures, six out of 13 participants demonstrated positive increases on the RBANS and subtests of the TEA (18, 36, 37). Nine out of the 15 participants demonstrated positive increases on the language scores as well (18, 25, 36–38).

In summary, the evidence shows that though metacognitive paradigms can be applied to language therapy for people with aphasia, the results are equivocal with only a little more than half of all participants benefitting overall. The varied outcomes can be explained by the heterogeneous samples of participants utilized between studies with vastly different designs, and only small sample sizes available for evaluation. Though the results demonstrate the viability of utilizing metacognitive treatment for people with aphasia, there is not yet enough evidence to conclude the benefits of such treatment in this population. See Supplementary Material D for further details on treatments utilized.

### How Outcomes in the TBI Literature Influence Potential Treatment for Persons With Aphasia?

The cogntive deficits that commonly follow TBI make metacognitive training an obvious choice for this population. Researchers tend to think of those with aphasia as having language disorder distinct from cognitive deficits, making metacogitive training appear to have less relevance. In fact, the relatively stronger cognitive skills may make those with aphasia stronger candidates. If memory and attention are less impaired, it follows that there is a higher likelihood of effectively using those skills to self-monitor language production.

The most commonly utilitzed outcome measures for testing cognitive skills as related to metacognition include attention and cognitive flexibility. Attention and cognitive flexibility, facets of executive function, which are closely related to metacognition, should be tested pre and post treatment in addition to target language outcomes. The treatment itself should utilize the breakdown of goals as seen in GMT, CO-OP and MSI as goal breakdown was observed in seven of the studies reporting positive results. Further, in order to foster self-awareness, verbal and video feedback should be utilized in addition to self-rating scales. In each of the studies that this was done, the effect was positive (14, 32). Using what has worked for individuals with TBI provides aphasiologists with the foundation needed to identify whether there is a therapeutic effect of metacognitive treatment for people with aphasia.

## Conclusion

Metacogitive treatment has proven to be efficacious for many individuals with brain injury and provides a potential new avenue of exploration for those recovering from aphasia. The treatment itself is meant to foster self-awareness and error awareness in individuals, thus increasing each individual's independence in their use of treatment techniques. Teaching the participant to break goals into manageable steps and recognize when errors occur will hopefully spill into their everyday life and lead to generalization of skills. The recognition of strengths and weakness, as well as breaking down of goals may be more efficacious for those with nonfluent aphasia, where recognition of errors may be more helpful to those with fluent aphasia. With that, the use of both strategies within therapy for every type of aphasia may lead to development of optimal metacognitive skills.

Researchers can deduce that the success of treatment itself highly relies on the individual's constitution and motivation toward achieving goals, not unlike other treatment methods. In the realm of research studies, most individuals are motivated as volunteers, although drop-outs and missing data occurs (12). Several questions remain including the appropriate dosage of treatment and the appropriate population (severity) of the individual being treated. For people with aphasia and some with TBI, there is also a question of how receptive language skills may interfere with learning and internalizing the breakdown of steps to reach set goals. Though these questions remain, further research on the feasibility and utility of metacognitive treatment in order to improve the functioning of individuals with brain injury, namely aphasia, should be completed.

## Future Directions

In the future, replication of studies is needed to validate the functionality and efficacy of metacognitive treatment for persons with ABI. Extant research shows that metacognitive treatment is useful, but the most effective treatment for different severity and presentations of persons with ABI remains unknown. Though studies included some individuals above the age of 65, we must focus research on the treatment practicality for persons over the age of 65, the ages where brain injury, namely stroke, is a common occurrence. Cognitive performance in the older population tends to decrease over time (67) so response to treatment is unknown for those above 65 years old.

Efficacy of metacognitive treatment for people with aphasia is not yet substantiated due to lack of evidence. There is also a lack of homogeneity amongst research studies, where different populations and different treatment paradigms were utilized. Hybrid treatment studies—those that involve both metacognitive treatment and language treatment– with a substantial number of participants need to be executed in order to begin determining whether metacognitive training is, in fact, appropriate for people with aphasia. Each study should focus on testing the metacognitive treatment protocol on different populations, starting with individuals with mild to moderate nonfluent aphasia, as the minimal evidence in this review show that this population responded adequately to metacognitive treatment. Studies should make use of information learned from this review, namely that treatment outcomes are likely to be improved when explicit education is provided to the participant in carrying out the various steps to complete desired outcomes. Video and verbal feedback should be considered for incorporation into future studies, as should self-awareness checklists to help increase an individual's awareness and independence while completing a task. In accordance with information gathered in this review, outcome measures should focus not only on language but on attention and cognitive flexibility as well. Following these guidelines, we will be able to take steps to discover the practicality and effectiveness of metacognitive treatment for people with aphasia.

## Data Availability Statement

The original contributions presented in the study are included in the article/Supplementary Material, further inquiries can be directed to the corresponding author.

## Author Contributions

All authors listed have made a substantial, direct, and intellectual contribution to the work and approved it for publication.

## Conflict of Interest

The authors declare that the research was conducted in the absence of any commercial or financial relationships that could be construed as a potential conflict of interest.

## Publisher's Note

All claims expressed in this article are solely those of the authors and do not necessarily represent those of their affiliated organizations, or those of the publisher, the editors and the reviewers. Any product that may be evaluated in this article, or claim that may be made by its manufacturer, is not guaranteed or endorsed by the publisher.
