# Supplementary material for: Metacognitive Treatment in Acquired Brain Injury and Its Applicability to Aphasia: A Systematic Review
Source: Front Rehabil Sci. 2022 Feb 4;3:813416. doi: 10.3389/fresc.2022.813416 (PMC9397955; doi:10.3389/fresc.2022.813416)
Supplement: Supplementary file 1 [file Data_Sheet_1.docx]

***Supplementary Material A***

MSI is based on the paradigm that the explicit teaching of one to self-regulate behavior will eventually lead the individual to internalize the skill of self-regulation (Cicerone & Giancino, 1992; Ramanathan et al., 2019; Finch et al., 2017). In MSI, individuals are taught a systematic method of breaking down complex goals into manageable steps and are then taught to self-monitor their performance throughout the completion of each step (Copley et al., 2015; Mayer et al., 2017, Ramanathan et al., 2019). The goal of MSI is to increase an individual’s self-awareness and internalize the ability to self-regulate, thus increasing one’s cognitive capabilities. Treatment dosage of MSI in the manuscripts reviewed, varied widely. For example, in Finch et al. (2017) participants received two hours of treatment per week for eight weeks (total= 16 hours). Copley et al. (2015) administered a total of five and a half hours of treatment per week for four weeks (total=22 hours).

The main tenant of GMT is instructing an individual to increase cognitive organization through increasing self-awareness of errors as well as self-regulation (Novakovic-Agopian et al. 2011; Tornas et al., 2016). Much like MSI, GMT focuses on being able to break down goals into manageable steps in order to support success in completion of goals. GMT protocol follows five steps to ensure adequate self-regulation: orienting an individual to the activity at hand, defining the goals for the given activity, listing steps to goal completion, learning to encode and recall steps of the target goal, and checking the outcome of the action as it compares to the target goal (Levine et al., 2000; Dawson et al., 2009). GMT utilizes a top-down treatment approach to promote an individual’s generalization of increased self-regulation and attention towards a goal. As with MSI, treatment dosages for GMT varied widely making it difficult to make comparisons across studies. For example, Levine et al. (2000) provided one hour of treatment just twice in their randomized control trial. Waid-Ebbs et al. (2014) provided treatment sessions two times per week for five weeks, where Tornas et al. (2016) provided eight two-hour sessions over four days.

CO-OP is based on Donald Meichanbaum’s argument that individuals can learn to regulate their behavior by setting a goal, making a plan, carrying out the plan and checking the success of the action (Dawson et al., 2009). CO-OP is client driven, where the focus lies on the individual’s ability to self-define the goal, identify breakdowns in their performance and self-evaluate the final outcome. CO-OP also highlights the importance of an individual’s support system’s involvement in the therapeutic process, as it helps with the transfer and generalization of skills (Dawson et al., 2009; Skidmore et al., 2011). Again, treatment dosages for CO-OP were variable. For example, Dawson et al. (2009) provided CO-OP treatment program to participants one-hour per day twice a week for ten weeks, where Skidmore et al. (2011) provided the program for forty-five minutes per day, five days per week for two weeks.

The feedback-based intervention utilized in Schmidt (2012) and Schmidt (2015) is a top-down therapeutic approach meant to increase an individual’s self-awareness throughout rehabilitation. The feedback-based intervention is generally utilized concurrently with another type of intervention, such as CRT or MSI. The intervention utilizes timely, specific, and consistent verbal and visual feedback in order to increase an individual’s self-awareness and self-knowledge. Schmidt et al. (2012) and Schmidt et al. (2015) provided up to four sessions of feedback treatment intervention over two weeks.

**Supplementary Material B**

Results from the Self-Regulation Skills Interview (SRSI), utilized in Toglia et al. (2010) and Goverover et al. (2007) indicated a clear trend towards increased use of strategies as well as statistically significant change in self-regulating behavior (F (1,19) = 9.63, p=.001), respectively. Results from the Behavior Rating Inventory of Executive Function for Adults (BRIEF-A), did not identify a trend in the study completed in Toglia et al. (2010). In Tornas et al. (2016) and Waid-Ebbs et al. (2014) the BRIEF-A did not reveal a significant positive change. Results from the Awareness Questionnaire (AQ) failed to identify any significant changes following treatment in three studies (Toglia et al., 2010; Goverover et al., 2007; Ownsworth et al., 2006). In Schmidt et al. (2012) and Schmidt et al. (2015), researchers observed a significant change in pre to post treatment AQ discrepancy scores for the verbal and video feedback experimental group. Results from the Dysexecutive Questionnaire (DEX), utilized to measure participant’s self-rating of cognition between pre- and post-treatment, demonstrated positive changes across three treatment studies (Dawson et al., 2009; Goodwin et al., 2016; Tornas et al., 2016). The questionnaires outlined above were the most frequently utilized across studies and identify trends seen across questionnaires throughout studies.

***Supplementary Material C***

Results from the Trails B, a test meant to measure visual scanning with distraction, identified positive changes across studies. For example, results from Raskin et al. (2019) showed significantly positive changes of Trails B. Novakovic-Agopian et al. (2011) reported that group A of their study, who received education followed by metacognitive training, also demonstrated significant changes on the Trails B (p=.007). Group B of the Novakovic-Agopian (2011) study did not demonstrate significant changes on the Trails B test. In Laatsch et al. (2004), the change was not significant but positive where the participant went from one error to no errors on the test.

Results from CPT-II demonstrated less sensitivity to cognitive changes following metacognitive rehabilitation. Tornas et al. (2016) identified significant improvement across time effect on the omission portion of the test; the t-test for the commission portion of the test approached significance. Lee and Sohlberg (2013) found that two of the four participants in their study demonstrated a significant t-score following metacognitive rehabilitation. The study conducted by Lee et al. (2018) demonstrated no statistically significant changes on either portion of the CPT-II (see Table 6).

The most frequently utilized measure of cognition following metacognitive rehabilitation was error frequency, where the outcome measure was used for five of the eighteen studies measuring cognition (Levine et al., 2000; Schmidt et al., 2012; Schmidt et al., 2015; Ownsworth et al., 2006; Ownsworth et al., 2010). Levine et al. (2000) measured error frequency through a proofreading task in both their RCT and single case study, as well as a meal preparation task in their single case study. The results showed that there were significant differences between groups in the RCT (one group receiving metacognitive rehabilitation and one group receiving motor skills training). The participant in the Levine et al. (2000) case study showed decreased errors in proofreading as well as significant decrease in errors in meal preparation. Schmidt (2012) and Schmidt (2015) also identified significant decrease in errors by the group receiving video and verbal feedback as compared to groups receiving experimental feedback. In a single case experimental study, the participant in Ownsworth et al. (2006) demonstrated a forty-four percent reduction in errors after receiving metacognitive rehabilitation. A subsequent study by Ownsworth et al. (2010) also showed positive results where participants demonstrated between thirteen percent and forty-five percent reduction of error behaviors following treatment.

***Supplementary Material D***

The four treatment types used to improve metacognition in aphasia include Intensive Cognitive-Communication Rehabiliatation Program (ICCR), Attention Process Training-II (APT-2), the Brain Budget Protocol and the Pragmatic Functional Paradigm. Gilmore et al. (2019) used ICCR in their quasi-experimental design study in which three of the participants, who were between forty-nine to ninety-seven TPO, had the diagnosis of aphasia. In ICCR, metacognitive rehabilitation was used across individual sessions and academic classes for college students with ABI. The participants had intensive therapy equaling three-hundred and sixty hours per semester over three semesters. The outcome measures included the Western Aphasia Battery-Revised (WAB-R), the Discourse Comprehension Test (DCT), the Scales of Cognitive and Communicative Ability for Neurorehabilitation (SCCAN), and the Repeatable Battery for the Assessment of Neuropsychological Status (RBANS). Results indicated positive gains on WAB-R and SCCAN from pre-treatment to post-treatment measures. One participant was deemed to have significant changes on the R-BANS and another participant demonstrated positive changes on the DCT following one semester of treatment. Overall, Gilmore et al. (2018) reported that students became more aware of strategies and accommodations that were beneficial to them following treatment.

Lee & Sohlberg (2013) carried out a single subject research design with four participants with aphasia who were eighteen to seventy-nine months post CVA. APT-3 in conjunction with metacognitive strategies was implemented thirty to forty-five minutes per week for eight weeks. Language outcome measures included AIMSWeb Maze Reading; cognitive outcome measures included CPT-II and the Test of Everyday Attention (TEA). Two of four of the participants demonstrated significant positive changes on the Maze Reading task with large effect sizes (See Table 7). All participants demonstrated increased scores on select measures of attention between the CPT-II assessment and the TEA. Greater improvement on all measures was seen for the two participants who demonstrated significantly better reading scores on the Maze task.

Lee et al. (2018) again utilized the hybrid APT-3 and metacognitive strategy treatment, this time for six participants with aphasia. Participants, who were nine to eighty months post stroke, participated in thirty to forty min treatment sessions six times per week for a total of eighteen to twenty-four hours of treatment. AIMSWeb Maze reading was utilized as a language outcome measure. Other measures included a series of subtests from the TEA, the Weschler Memory Scale-Third Edition (WMS-III), the Psycholinguistic Assessments of Language Processing in Aphasia (PALPA), in addition to CPT-II were utilized as cognitive outcomes. Upon visual inspection of the Maze reading task, change in baseline accuracy with introduction of treatment was seen for three of six participants; a significant aggregate effect size for all participants was observed. Significant changes in the TEA Map Search subtest as well as the PALPA Pointing Span for Noun-Verb Sequences were also observed. Positive changes in both language outcomes and select cognitive measures were observed in the study.

Mayer et al. (2017) implemented a hybrid treatment of MSI and traditional language therapy approach named the Brain Budget protocol. In this treatment the participant was required implement a budget to allocation of cognitive resources while undergoing speech and language therapy. No standardized tests were used as language outcome measures in this study, rather behavioral outcome measures of oral reading, verbal expression and written expression were utilized. Following treatment, positive changes were demonstrated in all language outcome measures with maintenance of skills seen in oral reading and verbal expression skills. Following intervention, the participant was able to pass his driver’s examination and reported he would start work part-time in the near future. Despite positive results, it is not clear whether gains were due to the protocol, the concomitant treatment he was receiving or possibly to spontaneous recovery since the participant was only four months post onset at the time of treatment.

Rosell-Clari & Hernandez-Sacristan (2017) performed a single case study with a female participant approximately two and half years post onset utilizing the Pragmatic Functional Paradigm treatment. Within this treatment metacognitive strategies are used to increase transfer between skills learned in therapy and skills utilized in everyday communication. There were no significant changes noted on the Boston Diagnostic Aphasia Examination (BDAE), though researchers identified decreased paraphasias and improvement in the naming task. Mixed results were seen on the MetaLanguage in Aphasia Assessment (MetAphAs) tasks where three out of six subtests identified significant changes following treatment.
