# Supplementary material for: Metacognitive Treatment in Acquired Brain Injury and Its Applicability to Aphasia: A Systematic Review
Source: Front Rehabil Sci. 2022 Feb 4;3:813416. doi: 10.3389/fresc.2022.813416 (PMC9397955; doi:10.3389/fresc.2022.813416)
Supplement: Supplementary file 2 [file Data_Sheet_2.docx]

Appendix I

ABI: Acquired Brain Injury

AAD: Assessment of Awareness of Disability (Tham et al., 1999)

AD: Awareness of Deficit

APT: Attention Process Training (Sohlberg & Mateer, 2010)

ASHA-FACS: American Speech, Language and Hearing Association’s Functional Assessment of Communication (Frattali et al., 1995)

ASHA Journals: American Speech, Language and Hearing Association’s Journals

AQ: Awareness Questionnaire (Sherer et al., 1998)

BADS: Behavioral Assessment of the Dysexecutive Syndrome (Wilson et al., 1996)

BDAE: Boston Diagnostic Aphasia Examination (Goodglass & Kaplan, 1983)

BRIEF-A: Behavior Rating Inventory of Executive Function- Adult Version (Roth et al., 2005)

BVMT-R: Brief Visual Memory Test-Revised (Benedict, 1997)

CASP: Child and Adolescent Scale of Participation (McDougall et al., 2013)

CFQ: Cognitive Failures Questionnaire (Broadbent et al, 1982)

CHI TBI: Closed Head Injury Traumatic Brain Injury

CIQ: Community Integration Questionnaire (Willer et al., 1994)

CPT: Connor’s Continuous Performance Task (Conners, 2000)

COMP: Canadian Occupational Performance Measure (Law et al., 1994)

CO-OP: Cognitive Orientation to Daily Occupational Performance

CRT: Cognitive Rehabilitation Therapy

CVA: Cerebral Vascular Accident

CWI: Color Word Interference Test (Delis et al., 2001)

DART- Dual-Task Attention Response Task (Dockree et al., 2006)

DASS: Depression Anxiety Stress Scale (Antony et al., 1998)

DCT: Discourse Comprehension Test (Brookshire & Nicholas, 1993)

DEX: Dysexecutive Questionnaire (Burgess et al., 1998)

DKEFS: Delis-Kaplan Executive Function System (Delis et al., 2001)

DPT: Discourse Processing Treatment

EAT: Error Awareness Task (Hester et al., 2005)

EMQ: Everyday Memory Questionnaire (Mateer et al., 1987)

FRsBE: Frontal Systems Behavior Scale (Grace & Malloy, 2002)

GAS: Goal Attainment Scaling (Kiresuk & Sherman, 1968)

GMT: Goal Management Training

HIV: Human Immunodeficiency Virus

HVLT-R: Hopkins Verbal Learning Test-Revised (Brandt, 1991)

IADLs: Independent Activities of Daily Living

ICCR: Intensive Cognitive-Communication Rehabilitation

IQ: Intelligence Quotient

LCQ: LaTrobe Communication Questionnaire (Douglas et al., 2000)

LLBA: Linguistics and Language Behavior Abstracts

MCLA: Measure of Cognitive Linguistic Abilities (Ellmo et al., 1995)

MEPSM: Means-Ends Problem Solving Measure (Spivack et al, 1976)

MET: Multiple Errands Test (Knight et al, 2002)

MetAphAs: MetaLanguage in Aphasia Assessment (Hernandez- Sacristan et al., 2012; Rosell-Clari et al., 2014)

MI: Metacomponential Interview (Clements & Nastasi, 1990)

MIST: Memory for Intentions Screening Test (Raskin, 2009)

MS: Multiple Sclerosis

MSI: Metacognitive Strategy Instruction

MST: Metacognitive Skills Training

Neuro-QOL: Neurologic Quality of Life (Gershon et al., 2012)

OZC: Oliver Zangwill Centre for Neuropsychological Rehabilitation

PALPA: Psycholinguistic Assessments of Language Processing in Aphasia (Kay et al., 1992)

PCRS: Patient Competency Rating Scale (Prigatano et al., 1986)

PEDro+: Physiotherapy Evidence Database Rating Scale-Plus

PPIC: Profile of Pragmatic Impairment in Communication (Linscott et al., 2003)

PHI TBI: Penetrating Head Injury Traumatic Brain Injury

PM: Prospective Memory

PMQ: Prospective Memory Questionnaire (Hannon et al., 1995)

PRISMA: Preferred Reporting Items for Systematic Reviews and Meta-Analysis Guidelines

RBANS: Repeatable Battery for Assessment of Neuropsychological Status (Randolph, 2012)

RMP: Raven’s Progressive Matrices (Raven et al., 2003)

RCT: Randomized Control Trial

SADI: Self-Awareness of Deficits Interview (Fleming et al., 1996)

SCANN: Scales of Cognitive and Communicative Ability for Neurorehabilitation (Holland & Milman, 2012)

SCED+: Single Case Experimental Design Scale-Plus

SPIRQ: Self-perceptions in Rehabilitation Questionnaire (Ownsworth et al., 2009)

SPSVM: Social Problem-Solving Video Measure (Kendall et al., 1997)

SRSI: Self-Regulation Skills Interview (Lundqvist et al. 2010; Ownsworth et al., 2000)

TEA: Test of Everyday Attention (Robertson et al., 1994)

TBI: Traumatic Brain Injury

TBI-QOL: Traumatic Brain Injury Quality of Life (Tulsky et al., 2016)

TMT: Trail Making Test (Delis et al., 2001; Reitan & Wolfson, 1985)

TOL: Tower of London (Berg et al., 2010)

TPO: Time Post Onset

UPSA: UCSD Performance Based Skills Assessment (Patterson et al., 2001)

VFT: Verbal Fluency Test (Delis et al., 2001)

WAB-R: Western Aphasia Battery-Revised (Kertesz, 2006)

WAIS-R: Weschler Adult Intelligence Scale-Revised (Weschler, 1995)

WCST: Wisconsin Card Sorting Test (Heaton et al., 1993)

WMS-R: Weschler’s Memory Scale-Revised (Weschler, 1990)

WHO-QOL: World Health Organization Quality of Life (WHOQOL Group, 1998)

References

Antony, M.M., Bieling, P.J., Cox, B.J., Enns, M.W., & Swinson, R.P. (1998). Psychometric

properties of the 42-item and 21-item versions of the Depression Anxiety Stress Scales (DASS) in clinical groups and a community sample. *Psychological Assessment, 10,* 176-181.

Benedict, R. (1997).  *Brief Visuospatial Memory Test-Revised.* Odessa, FL: Psychological

Assessment Resources.

Berg, W.K., Byrd, D.L., McNamara, J.P., & Case, K. (2010). Deconstructing the tower:

Parameters and predictors of problem difficulty on the Tower of London task.  *Brain & Cognition, 72*(3), 472-482.

Brandt, J. (1991). The Hopkins Verbal Learning Test: Development of a new memory test with

six equivalent forms.  *Clinical Neuropsychologist, 5,* 125-142.

Broadbent, D.E., Cooper, P.F., Fitzgerald, R., & Parkes, K.R. (1982). The cognitive failures

questionnaire (CFQ) and its correlates. *British Journal of Clinical Psychology, 21*(Pt 1), 1-16.

Brookshire, R.H. & Nicholas, L.E. (1993). *Discourse Comprehension Test.* Minneapolis, MN:

BRK Publishers.

Burgess, P.W., Alderman, N., Evans, J., Emslie, H. & Wilson, B.A. (1998). The evological

validity of tests of executive function. *Journal of the International Neuropsychological Society, 4,* 547-558.

Busk, P. L., & Serlin, R. C. (1992). Meta-analysis for single case research. In T. R. Kratochwill

& J. R. Levin (Eds.), *Single case research design and analysis* (pp. 197–198). Hillsdale, NJ: Lawrence Erlbaum Associates.

Clements, D.H., & Nastasi, B.K. (1990). Dynamic approach to measurement of children’s

metacomponential functioning. *Inteligence, 14*(1), 109-125.

Conners, C.K. (2000). *Conners’ continuous performance test II.* Toronto: Multi-Health Systems,

Inc.

Delis., D., Kaplan, E., & Kramer, J. (2001). *Delis-Kaplan Executive Function System.* San

Antonio, TX: Pearson Assessment and Information.

Dockree, P.J., Bellgrove, M.A., O’Keeffe, F.M., Moloney, P., Aimola, L., Carton, S., &

Robertson, I.H. (2006). Sustained attention in traumatic brain injury (TBI) and healthy controls: Enhanced sensitivity with dual-task load. *Experimental Brain Research, 168,* 218-229.

Douglas, J., Bracy, C., Snow, P. (2000). *La Trobe Communication Questionnaire.* LaTrobe

University.

Ellmo, W., Graser, J., Krchnavek, B., Hauck, K., Calabrese, D. (1995). *Measure of Cognitive*

*Linguistic Abilities (MCLA).*  USA: The Speech Bin.

Fleming, J., Strong, J., & Ashton, R. (1996). Self-awareness of deficits in adults with traumatic

brain injury: How best to measure? *Brain Injury, 10,* 1-15.

Frattali, C.M., Thompson, C.K., Holland, A.L., Wohl, C.B., & Ferketic, M.M. (1995). *The*

*American Speech-Language-Hearing Association Functional Assessment of Communication Skills for adults (ASHA FACS).* Rockville, MD: American Speech-Language-Hearing Association.

Gershon, R.C., Lai, J.S., Bode, R., Choi, S., Moy, C., Bleck, T., … Cella, D. (2012). Neuro-

QOL: Quality of life items banks for adults with neurological disorders: Item development and calibrations based upon clinica and general population testing. *Quality of Life Research, 21*(3), 475-486.

Goodglass, H. & Kaplan, E. (1983). *Boston Diagnostic Aphasia Examination.* Philadelphia, PA:

Lea & Febinger.

Grace, J., & Malloy, P.F. (2002).  *Frontal systems behavior scale. Professional manual.* Lutz,

FL: PAR.

Hannon, R., Adams, P., Harrington, S., & Fries-Dias, C. (1995). Effects of brain injury and age

on propective memory self-rating and performance. Rehabilitation Psychology, 40, 289*–*298.

Heaton, R.K., Chelune, G.J., Talley, J.L., Kay, G.G. & Curtiss, G. (1993). *Wisconsin Card*

*Sorting Test manual: Revised and expanded.* Odessa, FL: Psychological Assessment Resources.

Hernandez-Sacristan, C., Rosell-Clari, V., Serra-Alegre, E., & Quiles-Climent, J. (2012). On

natural metalinguistic abilities in aphasia. A preliminary study. *Aphasiology, 26*(2), 199-219.

Hester, R., Foxe, J.J., Molholm, S. Shpaner, M., & Garavan, H. (2005). Neural mechanisms

involved in error processing: A comparison of errors made with and without awareness. *Neuroimage, 27*(3), 602-608.

Holland, A.L. & Milman, L. (2012). *Scales of Cognitive and Communicative Ability for*

*Neurorehabilitation (SCCAN).* Austin, TX: Pro-Ed.

Kay, J., Lesser, R., & Coltheart, M. (1992). *Psycholinguistic assessment of language processing*

*in aphasia.* Bury St. Edmunds, UK: Thames Valley Test Company.

Kendall, E., Shum, D., Haslson, D., Bunning, S., & Teh, M. (1997). The assessment of social

problem-solving ability following traumatic brain injury. *Journal of Head Trauma Rehabilitation, 12*(3), 68-78.

Kertesz, A. (2006). *Western Aphasia Battery-Revised.* San Antonio, TX: PsychCorp.

Kiresuk, T.J. & Sherman, R.E.(1968). Goal attainment scaling: A general method for evaluating

comprehensive community mental health programs. *Community Mental Health Journal, 4*(6), 443-453.

Knight, C., Alderman, N., & Burgess, P.W. (2002). Development of simplified version of the

multiple errads test for use in hospital settings. *Neuropschological Rehabilitation, 12,* 231-255.

Law, M., Baptiste, S., Carswell, A., McColl, M. A., Polatajko, H., & Pollock, N. (1994).

*Canadian Occupational Performance Measure (2^nd^ ed.).* Toronto, ON: CAOT.

Linscott, R.J., Knight, R.G., Godfrey, H.P.D. (2003). *Profile of Pragmatic Impairment in*

*Communication.* University of Otago.

Lundqvist, A., Linnros, H., Orlenius, H., & Samuelsson, K. (2010). Improved self-awareness and

coping strategies for patients with acquired brain injury—A group therapy programme. *Brain Injury, 24*(6), 823-832.

Mateer, C., Sohlberg, M., & Crinean, J. (1987). Focus on clinical research: Perceptions of

memory function in individuals with closed-head injury. Journal of Head Trauma Rehabilitation, 2, 74*–*84.

McDougall, J., Bedell, G., & Wright, V. (2013). The youth report version of the Child and

Adolescent Scale of Participation (CASP): Assessment of psychometric properties and comparison with parent report. *Child: Care, Health and Development, 39*(4), 512-522.

Ownsworth, T., Fleming, J., Stewart, E., & Griffin, J. (2009). The self-perceptions in

rehabilitation questionnaire: A new measure of therapy progress in brain injury rehabilitation. Paper presented at 6^th^ Symposium on Neuropsychological Rehabilitation: Aug 3-4; Tallin, Estonia.

Ownsworth, T., McFarland, K., & Young, R.M. (2000). Development and standardization of the

self-regulation skills interview (SRSI): A new clinical assessment tool for acquired brain injury. *Clinical Neuropsychologist, 14,* 76-92.

Patterson, T.L., Goldman, S., McKibbin, C.L., Hughs, T., & Jeste, D.V. (2001). UCSD

performance-based assessment: Development of a new measure of everyday functioning for severely metally ill adults. *Schizophrenic Bulletin, 27,* 235-245.

Prigatano, G.P., Fordyce, D.J., &. Zeiner, H.K. (1986). *Neuropsychological rehabilitation after*

*brain injury.* Baltimore: John Hopkins University Press.

Raskin, S. A. (2009). Memory for intentions screening test: Psychometric properties and clinical

evidence. *Brain Impairment, 10,* 23-33.

Randolph, C. (2012). *Repeatable Battery for the Assessment of Neuropsychological Status*

*Update (RBANS).* Bloomington, MN: Pearson Clinical.

Raven, J., Raven, J.C., & Court, J.H. (2003). *Manual for Raven’s Progressive Matrices and*

*Vocabulary Scales.* San Antonio, TX: Harcourt Assessment.

Rees, L., Marshall, S., Hartridge, C., & Mackie, D.(2007). Cognitive interventions post cquired

brain injury. *Brain Injury*, *21*(2), 161–200. <https://doi.org/10.1080/02699050701201813>

Reitan, R. & Wolfson, D. (1985). *The Halstead-Reitan Neuropsychological Test Battery.*

Tucson, AZ: Neuropsychology Press.

Robertson, I. H., Ward, T., Ridgeway, V., & Nimmo-Smith, I. (1994). *The test of everyday*

*attention.*  Bury St. Edmunds, UK: Thames Valley Test Co.

Roth, R.M., Isquith, P.K., & Gioia, G.A. (2005). *Behavior Rating Inventory of Executive*

*Function-Adult Version.* Lutz, FL: Psychological Assessment Resources, Inc.

Sohlberg, M.M. & Mateer, C.A. (2010) *Attention process training-3.* Youngsville, NC: Lash &

Associated Publishing/Training, Inc.

Spivack, G., Platt, J.J., & Shure, M.B. (1976). *The problem solving approach to adjustment: A*

*guide to research and intervention.* San Francisco: Jossey-Bass.

Tham, K., Bernspang, B., & Fisher, A.G. (1999). Development of the assessment of awareness

of disability. *Scandanavian Journal of Occupational Therapy, 6*, 184-190.

Tulsky, D.S., Kisala, P.A., Victorson, D., Carlozzi, N., Bushnik, T., Sherer, M.,…Cella, D.

(2016). TBI-QOL: Development and calibration of item banks to measure patient reported outcomes following traumatic brain injury. *Journal of Head Trauma Rehabilitation, 31*(1), 40-51.

Weschler, D. (1985). *The Weschler Adult Intelligence Scale-Revised.* New York: Psychological

Corporation.

Weschler, D. (1990). *Weschler Memory Scale-Revised.* New York, NY: The Psychological

Corporation.

WHOQOL Group. (1998). *WHOQOL User Manual.* Division of Mental Health and Prevention

of Substance Abuse: World Health Organization.

Willer, B., Ottenbacher, K.J., & Coad, M.L. (1994). The community integration questionnaire: A

comparative examination. *American Journal of Physical Medicine and Rehabilitation, 73,* 103-111.

Wilson, B.A., Alderman, N., Burgess, P.W., Emslie, H., & Evans, J.J. (1996). *Behavioural*

*assessment of the dysexecutive syndrome: The manual.* London: Thames Valley Test Company.
